# Antimicrobial Peptides Prediction method based on sequence multidimensional feature embedding

**DOI:** 10.3389/fgene.2022.1069558

**Published:** 2022-11-17

**Authors:** Benzhi Dong, Mengna Li, Bei Jiang, Bo Gao, Dan Li, Tianjiao Zhang

**Affiliations:** ^1^ College of Information and Computer Engineering, Northeast Forestry University, Harbin, China; ^2^ Tianjin Second People’s Hospital, Tianjin Institute of Hepatology, Tianjin, China; ^3^ Department of Radiology, The Second Affiliated Hospital of Harbin Medical University, Harbin, China

**Keywords:** deep learning, feature encoding, feature embedding, N-gram encoding, antimicrobial peptides

## Abstract

Antimicrobial peptides (AMPs) are alkaline substances with efficient bactericidal activity produced in living organisms. As the best substitute for antibiotics, they have been paid more and more attention in scientific research and clinical application. AMPs can be produced from almost all organisms and are capable of killing a wide variety of pathogenic microorganisms. In addition to being antibacterial, natural AMPs have many other therapeutically important activities, such as wound healing, antioxidant and immunomodulatory effects. To discover new AMPs, the use of wet experimental methods is expensive and difficult, and bioinformatics technology can effectively solve this problem. Recently, some deep learning methods have been applied to the prediction of AMPs and achieved good results. To further improve the prediction accuracy of AMPs, this paper designs a new deep learning method based on sequence multidimensional representation. By encoding and embedding sequence features, and then inputting the model to identify AMPs, high-precision classification of AMPs and Non-AMPs with lengths of 10–200 is achieved. The results show that our method improved accuracy by 1.05% compared to the most advanced model in independent data validation without decreasing other indicators.

## 1 Introduction

Antimicrobial peptides (AMPs) are host defense molecules produced by the innate immune system in a variety of organisms and have many advantages, such as rapid killing, low toxicity, and broad activity (Fjell et al., 2009), and their drug resistance is relatively low. About 50% of the amino acids in AMP are hydrophobic, and they can adopt an amphiphilic structure, which enables them to interact with and penetrate cell membranes, which then lead to disruption of membrane potential, changes in membrane permeability, and permeation of metabolites leakage, eventually leading to bacterial cell death (Kumar et al., 2018). AMPs not only exhibit synergy with antibiotics, but may also synergize with the immune system (Pasupuleti et al., 2012). At present, there are corresponding drug-resistant pathogenic strains of conventional antibiotics, and the drug-resistant problem of pathogenic bacteria has increasingly threatened people’s health. Finding new antibiotics is an effective way to solve the drug-resistant problem. The characteristics of high antibacterial activity, broad antibacterial spectrum, and wide selection range are considered to be an effective way to solve the problem of drug resistance (Hancock and Sahl, 2006). Given the multiple advantages of AMPs, there is an urgent need to identify new AMPs.

In recent years, the rapid development of bioinformatics has provided a rational design method for the acquisition of AMPs. We can predict AMPs based on their sequence information. At present, the research on sequence classification algorithms mainly focuses on the combination of classification algorithms and biological sequence features. Various applied machine learning models have also been applied in AMPs prediction, for example, support vector machines (SVM) (Lata et al., 2010; Meher et al., 2017; Agrawal et al., 2018; Gong et al., 2021; Zou et al., 2021; Zhang Q. et al., 2022), random forest (RF) (Bhadra et al., 2018; Veltri, 2015; Nakayama et al., 2021; Yang et al., 2021; Ao et al., 2022; Lv et al., 2022a), discriminant analysis (DA) (Thomas et al., 2010; Waghu et al., 2016), Hidden Markov (Fjell et al., 2009), k-nearest neighbors (Xiao et al., 2013), *etc.* The core problem of such methods is how to perform feature extraction on protein sequences, which is greatly affected by the feature extraction method, which limits the maximum performance of the model. In addition, artificial feature engineering is often required when machine learning builds a classification model. In this process, important information is likely to be lost. Deep learning methods that have developed rapidly in recent years can effectively solve this problem.

Deep learning methods can automatically learn features from the raw data through convolution operations, avoiding the loss of data features. Various deep learning methods have been applied in protein sequence classification, such as bidirectional long short-term memory network (Bi-LSTM) (Tng et al., 2021; Zhang Y. et al., 2022; Zhang et al., 2022c; Li et al., 2022; Qiao et al., 2022; Wang et al., 2022), two-dimensional convolutional neural network (2D CNN) (Le et al., 2021), deep residual network (ResNet) (Xu et al., 2021), graph convolutional network (GCN) (Chen et al., 2021), deep neural network (DNN) (Gao et al., 2019; Han et al., 2019; Le et al., 2019; Hathaway et al., 2021), and Recurrent Neural Network (RNN) (Zheng et al., 2020; Yun et al., 2021). These research methods have generally achieved good classification results and have attracted increasing attention. In the prediction of AMPs, deep learning methods have also received attention, such as deep neural network (DNN) (Veltri et al., 2018; Su et al., 2019; Fu et al., 2020; Yan et al., 2020), bidirectional long short-term memory network (Bi-LSTM) (Sharma et al., 2021a; Xiao et al., 2021; Sharma et al., 2022), and Transformer (Zhang et al., 2021). These models all demonstrate the superiority of deep learning in AMPs prediction.

Whether it is a machine learning method or a deep learning method, the first step of these methods is to represent protein sequences as machine-readable and to encode biological sequences with features, that is, to map biological sequences to digital sequences using digital signal processing methods. It is widely used in biological sequence classification. As an important biological sequence analysis method, biological sequence encoding has been studied by many scholars, for example, the interaction of protein sequences (Moretta et al., 2020; Khabbaz et al., 2021; Wani et al., 2021; Söylemez et al., 2022), sparse coding (binary coding) (Spänig and Heider, 2019; Akbar et al., 2021; Jain et al., 2021; Ren et al., 2022). In addition, pre-trained models in natural language processing (NLP) have been used in protein sequence analysis, for example, the word2vec method (Zhang et al., 2019; Dao et al., 2021) and the N-gram method (Li et al., 2018; Wu and Yu, 2021) showed excellent performance in prediction.

**FIGURE 1:**
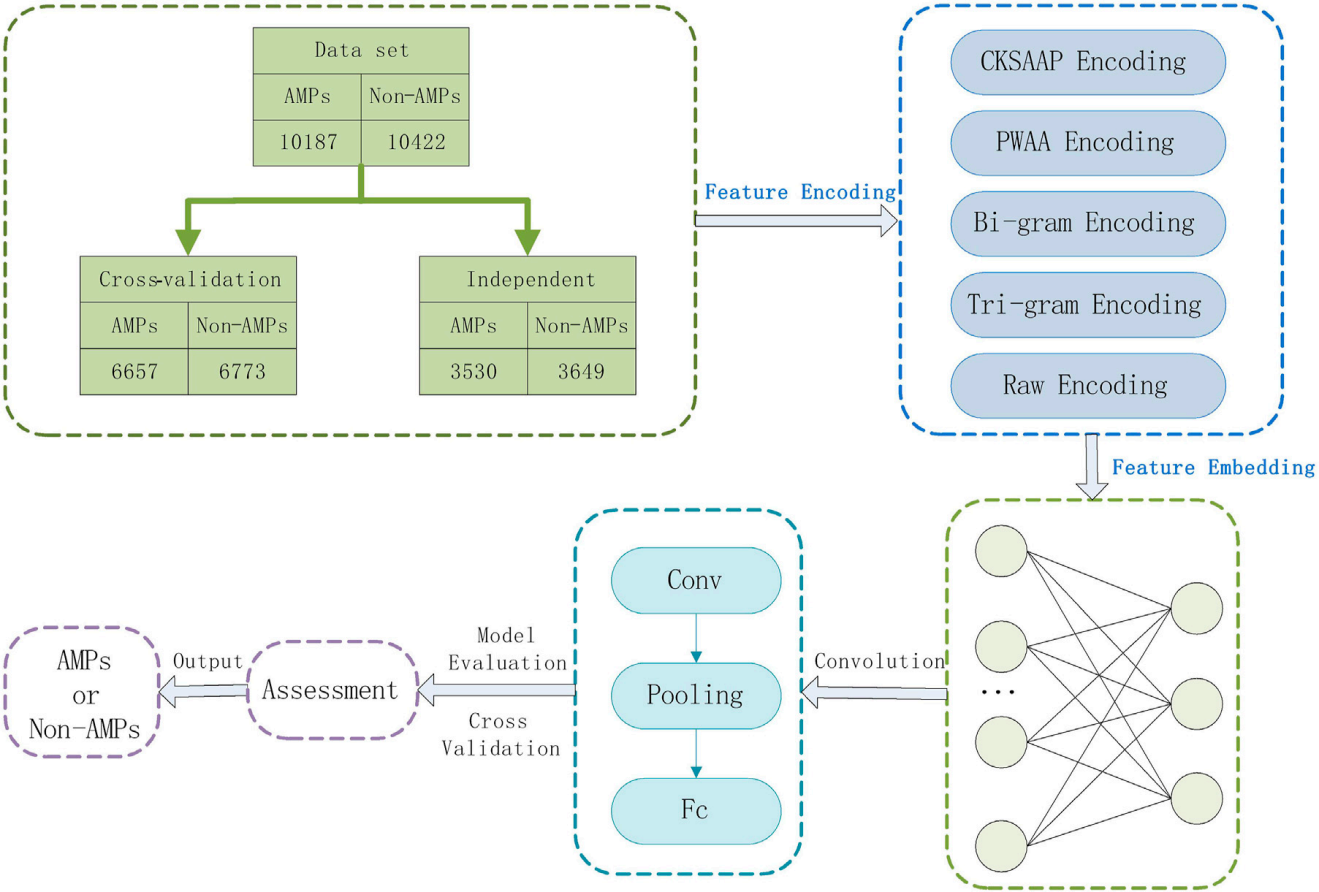
The workflow.

The AMPs classification methods are usually based on machine learning or deep learning consider the interaction between protein sequences and represents the sequences as a matrix, ignoring the upstream and downstream information of the sequences, and the prediction accuracy will be reduced during the classification process. In this paper, deep learning-based feature combinations of N-gram encoding, K-space amino acid pair composition (CKSAAP), position-weighted amino acid composition (PWAA), and raw sequence number encoding were selected to predict AMPs. The CKSAAP encoding effectively describes the short-range interactions between amino acids, the PWAA encoding determines the positional information of amino acids in the protein sequence, and considers the upstream and downstream information of the protein sequence, and the N-gram encoding enhances the expression of the protein sequence and reduces the training process. Information is lost. It not only considers the interaction and positional weight of amino acids in the protein sequence but also combines the upstream and downstream information in the sequence and enhances the expression of the AMPs sequence, avoiding the above problems and improving the prediction performance. To evaluate the model, we use a 10-fold cross-validation method. Figure 1 shows our workflow.

## 2 Materials and methods

### 2.1 Baseline datasets

In this study, we used the dataset of (Sharma et al., 2021b), which collected AMPs data belonging to 13 phyla and 41 kingdoms (animal kingdom) categories from NCBI and StarPepDB databases and obtained Non-AMPs data from the UniProt database. This dataset considers all AMPs of suitable length in the animal kingdom to train the model. After the data is de-redundant, the dataset finally consists of 10,187 AMPs and 10,422 Non-AMPs, shown in supplementary materialthe, which contains about 65% of AMPs and non-AMPs. AMPs were used as the cross-validation dataset to train our model, and the rest contained about 35% of AMPs and non-AMPs as independent datasets for evaluating model performance, whose composition is shown in Table 1.

**TABLE 1:** Statistics for datasets.

	Total	Cross-validation	Independent
AMPs	10,187	6,657	3,530
Non-AMPs	10,422	6,773	3,649

### 2.2 Encoding method of sequence

#### 2.2.1 Raw sequence encoding

Protein is composed of 20 kinds of amino acids, each amino acid is represented by a character, and the sequences represented by these 20 kinds of characters contain important biological genetic information. The raw sequence encoding, that is, mapping the sequence to a set of numbers, reflects the selection bias of the AMPs sequence at each amino acid position. If given a protein sequence of length n, $S=\left(s_{1},\;s_{2},\;\ldots ,\;s_{n}\right)$ , where $s_{i}\in \left\{\;A,\;R,\;N,\;D,\;C,\;Q,\;E,\;G,\;H,\;I,\;L,\;K,\;M,\;F,\;P,\;S,\;T,\;W,\;Y,\;V\;\right\}$ , i=1,2,…,n , then the sequence S can be expressed as a one-dimensional vector of length n. For example, a protein sequence FLPKLFAKITKKNMAHIRC with a length of 19 can be used as a vector ${\left[5,\;10,\;13,\;9,\;10,\;5,\;1,\;9,\;8,\;17,\;9,\;9,\;12,\;11,\;1,\;7,\;8,\;15,\;2\right]}_{19}$ . The maximum length of protein sequences in the dataset used in this paper is 200, so we set the sequence coding dimension to 200, and all sequences shorter than 200 are filled with 0 at the end.

#### 2.2.2 Composition of k-space amino acid pairs (CKSAAP) encoding

CKSAAP is a coding scheme based on the interaction between amino acid pairs, which has been widely used in protein prediction (Yuan et al., 2022). CKSAAP can represent amino acids as a combination of multiple amino acid pairs with spacing K (Chen et al., 2011), reflecting the short-range interaction between amino acid pairs. If K = 0, there are 400 residue pairs with spacing 0 (AA, AC, AD, AE, …, YY). The eigenvector can be calculated by Eq. 1:

$${\left(\frac{N_{AA}}{N_{Total}},\frac{N_{AC}}{N_{Total}},\frac{N_{AD}}{N_{Total}},\frac{N_{AE}}{N_{Total}},\ldots ,\frac{N_{YY}}{N_{Total}}\right)}_{400}$$ (1)

Where, N_Total_ = L-K-1, N_Total_ represents the total number of residue pairs in the protein sequence, L represents the sequence length, and K represents the amino acid spacing. For example, when the sequence length is 200 and K = 0, 1, 2, 3, the values of N_Total_ are 199, 198, 197, and 196. In this paper, we take K as 0, 1, 2, 3, 4, and 5, so the total dimension of this feature is 2,400.

#### 2.2.3 Position weighted amino acid composition (PWAA) encoding

To determine the position information of amino acids in the protein sequences, we used the PWAA method for encoding. Given amino acid residue a_i_ (i = 1, 2, 3,..., 20), we can calculate the positional information of a_i_ in a protein sequence by Eq. 2:

$$C_{i}=\frac{1}{L\left(L+1\right)}\overset{L}{\underset{j=-L}{\sum }}x_{i,j}\left(j+\frac{\left|j\right|}{L}\right)\left(j=-L,\ldots ,q,\ldots ,L\right)$$ (2)

Where L represents the data of upstream residue or downstream residue at the central site of the protein sequence fragment, if a_i_ is the residue at the *j*th position of the protein sequence fragment, then x (i, j) = 1, otherwise x (i, j) = 0. Generally, the closer a_i_ is to the center position (position 0), The smaller the absolute value of C_i_. The PWAA encoding involves 20 kinds of amino acid residues, so this method encodes a dimension of 20.

#### 2.2.4 N-gram encoding

**FIGURE 2:**
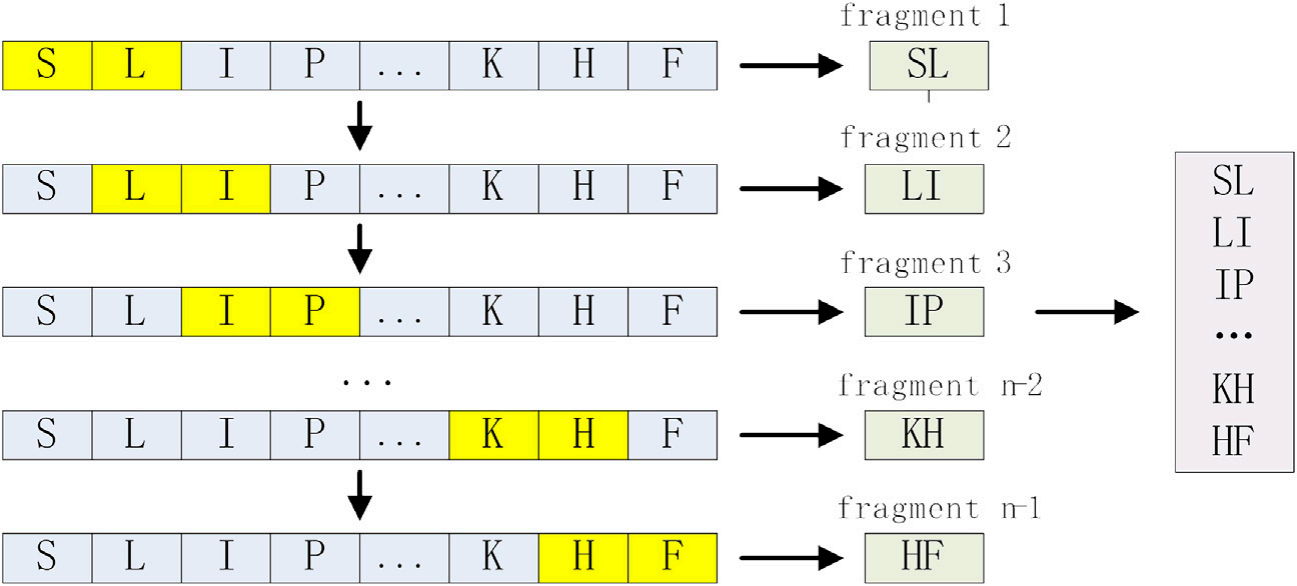
Bi-gram encoding process.

N-gram is a statistical language model, which can be applied to protein sequence analysis to enhance the expression of protein sequences (Sharma and Srivastava, 2021). We treat each amino acid residue of a protein sequence of length L-N+1 as a word and each sequence as a sentence. In this study, our data length is short, and the Bi-gram (binary model) and tri-gram (ternary model) we used are enough to enhance the expression of AMPs sequences. For an raw sequence of length n S= (s_1_, s_2_, … s_n_), Bi-gram can be expressed as S_2_=(s_1_s_2_,s_2_s_3_, … ,s_(n-1)_s_n_), whose length is n-1, and the coding process is shown in Figure 2. Similarly, Tri-gram can be expressed as S_3_=(s_1_s_2_s_3_,s_2_s_3_s_4_, … ,s_(n-2)_s_(n-1)_s_n_), whose length is n-2. To align the encoding length of the N-gram, we set the encoding length of the N-gram to 200, and the encodings shorter than 200 are padded with 0 at the end, so the dimensions of the Bi-gram and Tri-gram are 200 respectively.

### 2.3 Deep learning model

**FIGURE 3:**
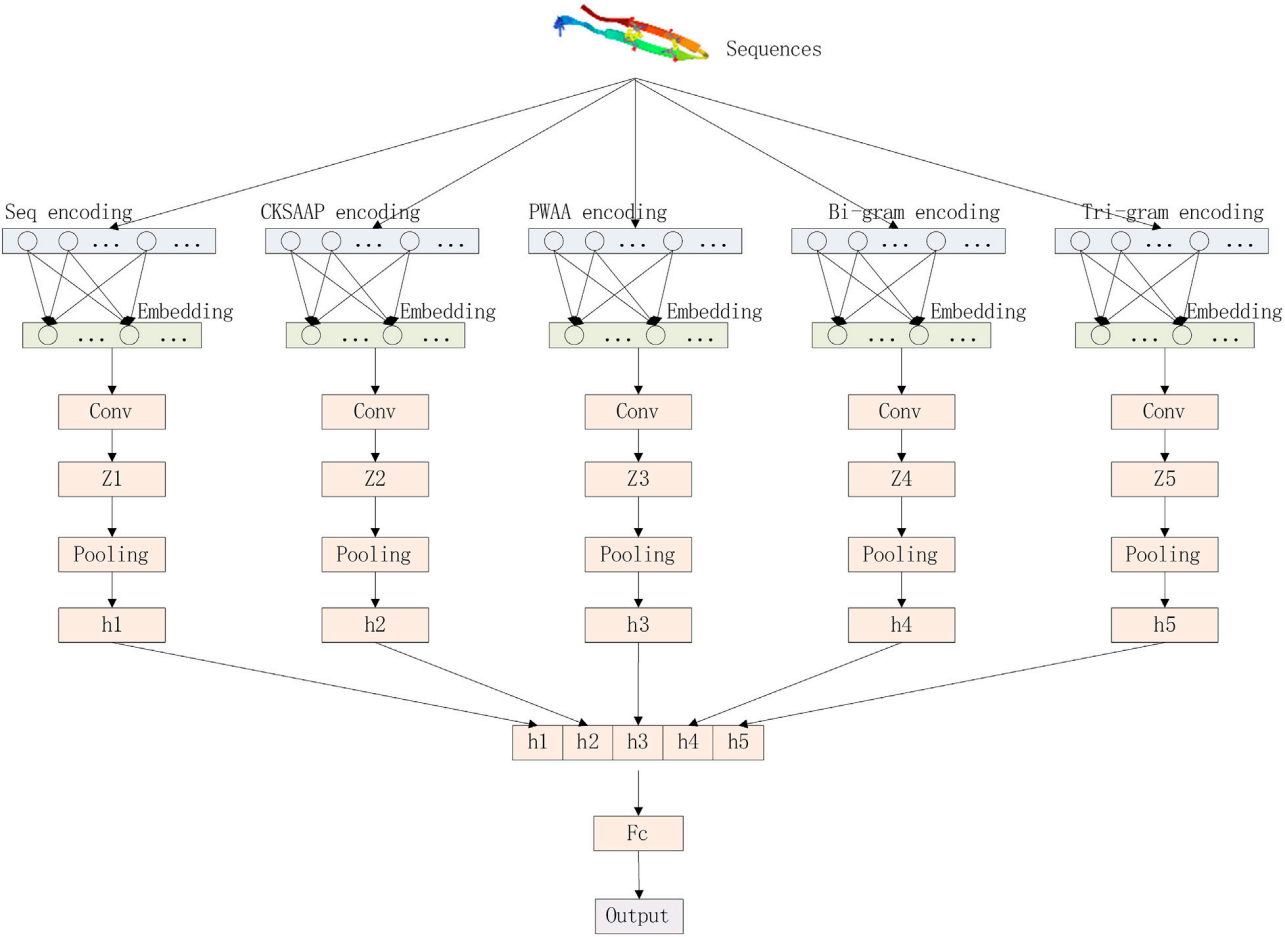
Model architecture diagram.

Our deep learning model consists of three parts: encoding layer, embedding layer, and convolutional layer. The model architecture is shown in Figure 3.

We convert protein sequences into numerical vectors using CKSAAP, PWAA, N-grams, and the numerical encoding of the raw sequence and then pass these vectors into the embedding layer. The embedding layer converts the sparse vector into a dense vector and reduces the dimension of the vector to facilitate the processing of the upper neural network. The processing process of the embedding layer can be represented by the following matrix operations. The first matrix represents the input feature matrix, the middle matrix represents the weight of this layer, and the multiplied result matrix represents the dimension-reduced feature matrix.

$$\left[\begin{array}{ccc} 0 & 1 & \begin{array}{cc} 0 & 0 \end{array} \end{array}\right]\times \left[\begin{array}{ccc} \begin{array}{c} 2\\ 4 \end{array} & \begin{array}{c} 5\\ 2 \end{array} & \begin{array}{c} 7\\ 1 \end{array}\\ 2 & 8 & 5\\ 3 & 6 & 8 \end{array}\right]=\left[\begin{array}{ccc} 4 & 2 & 1 \end{array}\right]$$

The convolution layer convolutes the embedded matrix E with N parallel convolution blocks, which can be composed of a set of triples {(s_k_, q_k_, r_k_)}_(k=1,..., N)_, where s_K_ represents the size of the convolution filter, q_k_ represents the number of convolution filters in the convolution block, and r_k_ represents the activation function corresponding to the convolution block. The convolution direction is one-dimensional convolution along the direction of the sequence, and the convolution block will output a set of feature maps ${\left\{Z_{k}\in R^{\left(l-s_{k}+1\right)\times q_{k}}\right\}}_{k=1,\;\ldots ,\;N}$ , the convolution block k can be expressed by Eq. 3:

$$Z_{k}\left(m,\;q\right)=a_{k}\left(\sum_{i=0}^{e}\sum_{j=0}^{s_{k}}C\left(i,j,k\right)\times E\left(i,m+j\right)\right)$$ (3)

Where, q = 1, … , q_k_, C∈ $R^{e\times s_{k}\times q_{k}}$ contains the weight tensors of all q_k_ convolution filters in this convolution block. a_k_ is the activation function, and we use Rectified Linear Unit (ReLU) as the activation. Z_k_ (m, q) is the feature map Z_k_ of the (m, q)th element in the training phase.

Global average pooling integrates global spatial information, while CKSAAP and PWAA codes encode protein sequences as sparse matrices (with many 0s). Choosing global average pooling may reduce the accuracy of prediction, while global pooling can preserve more Boundary information. Therefore, after obtaining each feature map, we perform a global maximum pooling operation to reduce the number of features in the training phase to prevent overfitting. The vector h_k_ can be calculated by Eq. 4:

$$h_{k}=\left[\max Z_{k}\left(:,\;1\right);\max Z_{k}\left(:,\;2\right);\ldots ;\max Z_{k}\left(:,\;q_{k}\right)\right]$$ (4)

Finally, the vector h = [h_1_; h_2_; …; h_N_] is obtained by fully connecting all h_k_, and the prediction results are output.

Because the learning rate is greatly affected by the output error, the cross-entropy loss function has a larger parameter adjustment range in the early stage of model training, which can make the model training converge faster. To improve the classification efficiency, we use the binary cross-entropy function as our loss function, which can be expressed by Eq. 5:

$$Loss=-\frac{1}{N}\overset{N}{\underset{i=1}{\sum }}y_{i}\times \log\left(p\left(y_{i}\right)\right)+\left(1-y_{i}\right)\times \log\left(1-p\left(y_{i}\right)\right)$$ (5)

Where, y represents the binary label 0 or 1, and p(y) represents the probability that the output belongs to the y label. If the predicted value p(y) approaches 1, then the value of the loss function should approach 0. Conversely, if the predicted value p(y) approaches 0 at this point, the value of the loss function should be very large.

### 2.4 Model evaluation

To objectively evaluate the performance of this method, we train the model using a 10-fold cross-validation method, which randomly divides the negative and positive samples into k (k = 10) equal-sized subsamples. Among the k subsamples, one sub-sample is reserved as validation data for testing the model, and the remaining k-1 subsamples are used as training data (Lv et al., 2022b; Zhang et al., 2022d). Then repeat the cross-validation process for K (k = 10) times (folds), and each sub-sample is used only once as validation data.

To evaluate the precision of the results, we use 7 metrics of accuracy (A_cc_), sensitivity (S_n_), precision (P_r_), specificity (S_p_), F1 score (F_s_), balance accuracy (B_a_), and area under the curve (AUROC) on independent datasets, as shown in Formulas 6 to 12.

$$A_{\text{cc}}=\frac{\mathrm{TP}+\mathrm{TN}}{\mathrm{TP}+\mathrm{FN}+\mathrm{TN}+\mathrm{FP}}$$ (6)

$$S_{n}=\frac{\mathrm{TP}}{\mathrm{TP}+\mathrm{FN}}$$ (7)

$$P_{r}=\frac{\mathrm{TP}}{\mathrm{TP}+\mathrm{FP}}$$ (8)

$$S_{p}=\frac{\mathrm{TN}}{\mathrm{TN}+\mathrm{FP}}$$ (9)

$$F_{s}=\frac{2\times S_{n}\times P_{r}}{\mathrm{TN}+\mathrm{FP}}$$ (10)

$$B_{a}=\frac{S_{n}+P_{r}}{2}$$ (11)

AUROC=∫TPR d(FPR) (12)

Where, TP is the true positive, FP is the false positive, TN is the true negative, FN is the false negative, TPR is the true positive and FPR is the false positive.

## 3 Results and discussion

### 3.1 Sequence composition analysis based on benchmark datasets

**FIGURE 4:**
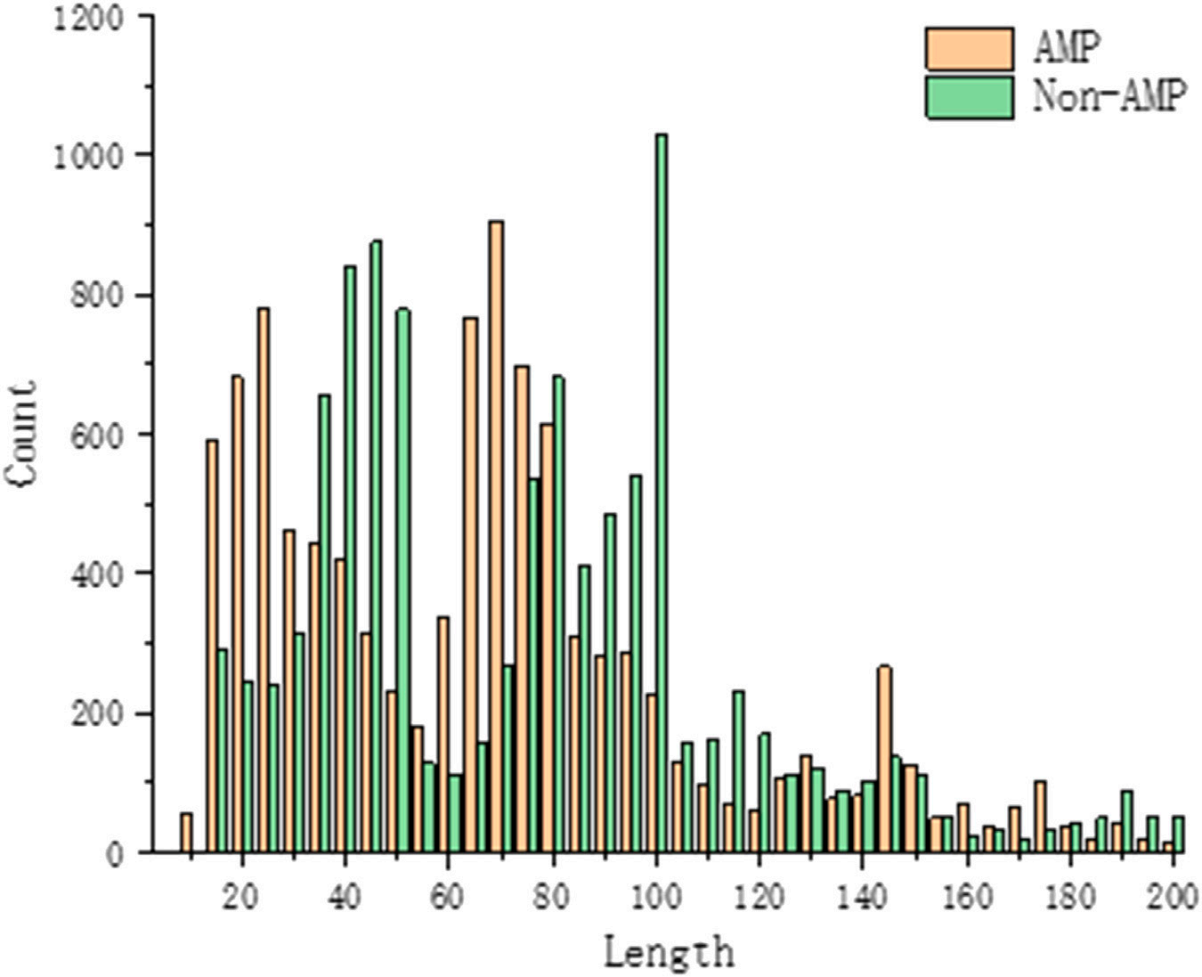
Benchmark dataset protein sequence length statistics.

All proteins are made up of 20 amino acid residues, but the frequency of amino acid residues in each protein varies and the lengths of the amino acid sequences that make up the protein vary. During model training, the composition of peptides in the benchmark dataset is very important to analyze the properties of antimicrobial peptides. By counting the centralized peptide lengths of the AMPs and Non-AMPs data, the peptide lengths of our AMPs and Non-AMPs data sets are between 10 and 200, and most of the peptides are below 100 in length, as shown in Figure 4.

**FIGURE 5:**
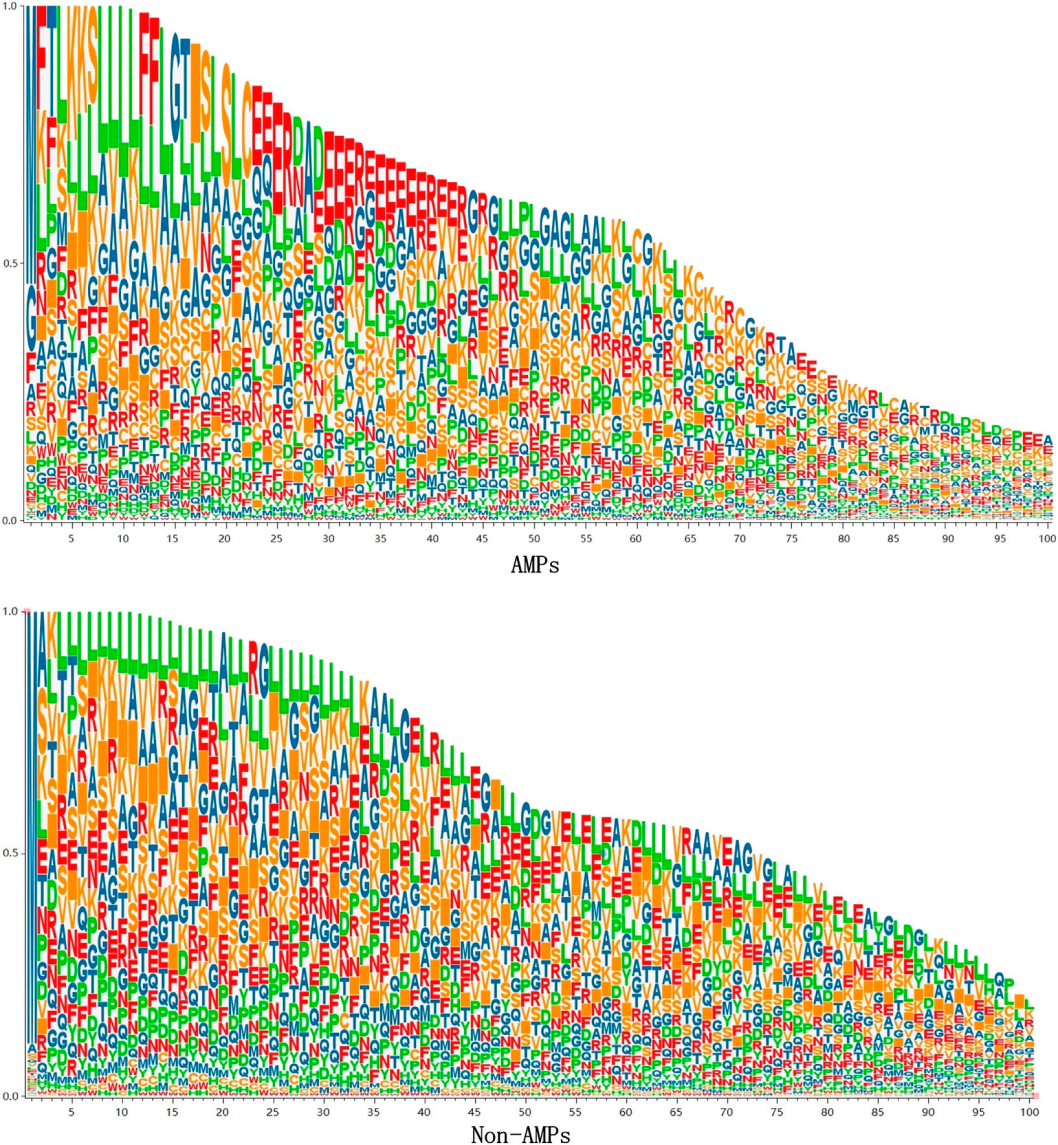
sequence logo diagram.

To analyze the sequence consisting of the benchmark dataset, we counted the occurrence frequency of different amino acids at each sequence position. Since the length of AMPs sequences is mainly concentrated in 10–100, we only draw the sequence logo diagram of the first 100 positions, as shown in Figure 5. It can be seen from the figure that specific amino acids belonging to AMPs and Non-AMPs have different positional preferences. In the AMPs sequence, the positions 22–42 are often occupied by glutamic acid (E), and in the Non-AMPs sequence, the positions 22–42 are often occupied by glutamic acid (E). The positions 4–33 are often occupied by leucine (L), and this difference may be due to their belonging to different protein families.

### 3.2 Comparison of feature coding methods for different combinations

To study the prediction effect of different feature encodings, we conducted experiments on the combination of these three feature encodings with the original sequences based on the verification set. We treat the Bi-gram and Tri-gram encodings as independent feature encoding methods, and finally, combine all the features for experiments, so we did five sets of comparative experiments. CKSAAP encoding and PWAA encoding only extract amino acid combination and position information. The feature encoding is a sparse matrix with many 0 elements. When it is used alone, the prediction accuracy is relatively low, so the original sequence encoding is added to the experiment to make up. The experimental results are shown in Table 2.

**TABLE 2:** Comparison of different combination feature coding methods.

Coding	Acc(%)	Sn(%)	Pr (%)	Sp(%)	Fs(%)	Ba (%)	AUROC(%)
Seq + CKSAAP	96.36	97.34	95.36	95.42	96.34	96.38	99.38
Seq + PWAA	95.10	98.20	92.66	91.86	95.35	95.03	99.17
Seq + Bi-gram	97.57	98.48	96.83	96.62	97.65	97.55	99.64
Seq + Tri-gram	96.94	98.11	95.73	95.83	96.90	96.97	99.49
Seq + CKSAAP + PWAA + Bi-gram + Tri-gram	**98.11**	**99.15**	**97.21**	**97.02**	**98.17**	**98.08**	**99.74**

Note: the best performance on a metric is marked in bold.

**FIGURE 6:**
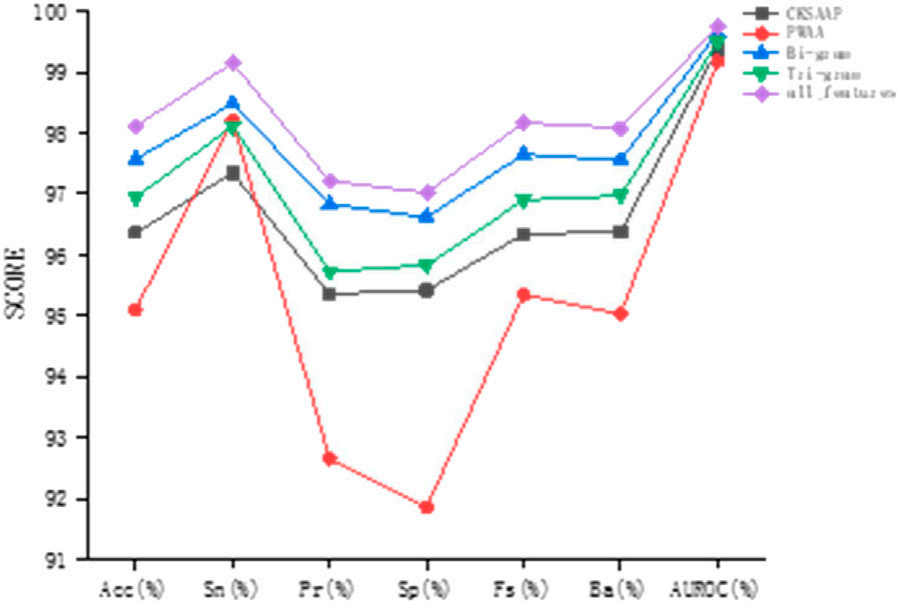
Comparison of different combination feature coding methods.

It can be found by observation that in the combination with the original sequence, Bi-gram encoding has the best prediction effect, and the sizes of various indicators after combination are most similar to Bi-gram encoding. Bi-gram encoding combines two adjacent amino acids to enhance sequence expression. Compared with Tri-gram encoding, Bi-gram encoding has stronger local association expression. PWAA encoding has the worst prediction effect and the various indicators are not as balanced as the other three encoding methods. This encoding method considers the upstream and downstream information of the sequence and does not consider the interaction between amino acids. It has only 20 dimensions and is a sparse matrix, which contains data Relatively few, even if there is a supplementary prediction effect encoded by the raw sequence, the effect is not good enough. CKSAAP encoding describes short-range interactions between amino acids. Although its form is also a sparse encoding, it has higher dimensions and more information, so the prediction effect is better than PWAA encoding. The prediction results of this study are most affected by Bi-gram encoding and less affected by PWAA encoding. After we combine these kinds of codes, the prediction effect is improved. As can be seen from Figure 6, this feature combination combines the advantages of these kinds of feature codes and considers the interaction of amino acids in protein sequences, position weights, and upstream and downstream information. And it is not affected by the imbalance of PWAA encoding indicators.

**FIGURE 7:**
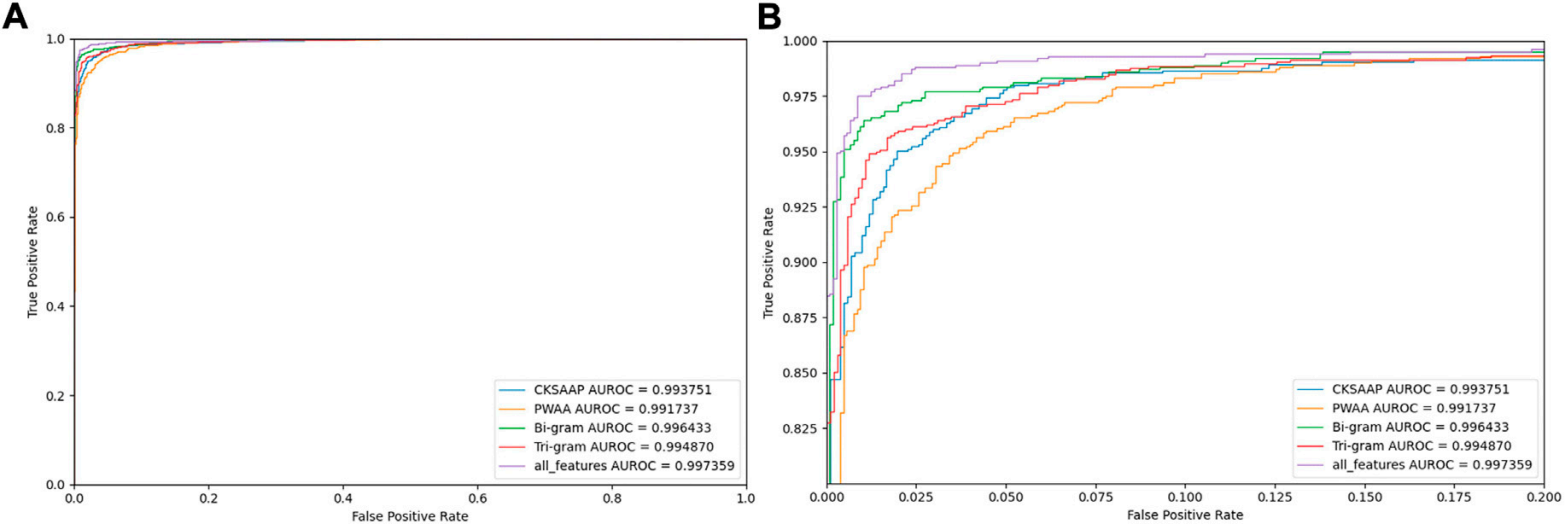
Comparing ROC curves with different feature codes. Note: **(B)** is a partially enlarged view of **(A)**.

To judge the recognition ability of various encoding combinations for AMPs, we plotted the ROC curves of various combinations, as shown in Figure 7.

### 3.3 Comparison with other methods

**FIGURE 8:**
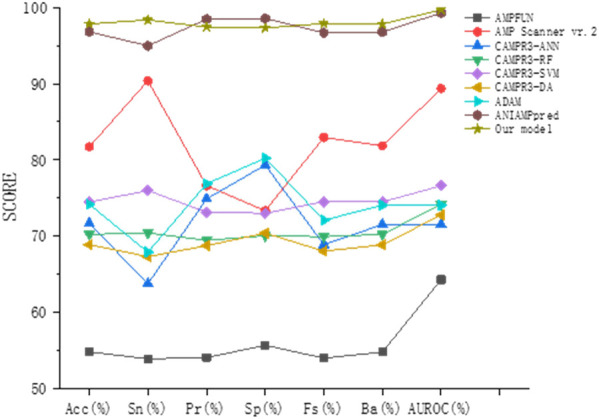
Performance comparison of different models.

To prove the effectiveness of our method, we compared the prediction results of the method proposed in this paper with other most advanced models (AMPFUN (Chung et al., 2020), AMP Scanner vr.2 (Veltri et al., 2018), CAMPR3 (Waghu et al., 2016), ADAM (Lee et al., 2015), ANIAMPpred (Sharma et al., 2021b)) based on independent test sets. The results are shown in Table 3 and Figure 8. It can be seen from the figure that the performance of ANIAMPpred and the method proposed in this paper is far superior to other models. In terms of PR and SP indicators, ANIAMPpred is slightly higher than our method, but we are the highest in other indicators. The accuracy of our model is 1.05% higher than that of the most advanced method.

**TABLE 3:** Performance comparison of different models.

Methods	Acc(%)	Sn(%)	Pr (%)	Sp(%)	Fs(%)	Ba (%)	AUROC(%)
AMPFUN	54.76	53.85	54.01	55.63	53.93	54.74	64.26
AMP Scanner vr.2	81.71	90.40	76.61	73.31	82.94	81.85	89.37
CAMPR3-ANN	71.64	63.71	74.87	79.31	68.84	71.51	71.51
CAMPR3-RF	70.20	70.40	69.43	70.02	69.91	70.21	74.15
CAMPR3-SVM	74.45	75.98	73.12	72.98	74.52	74.48	76.60
CAMPR3-DA	68.85	67.28	68.72	70.38	67.99	68.83	72.75
ADAM	74.15	67.85	76.86	80.24	72.07	74.04	74.04
ANIAMPpred	96.82	94.99	**98.50**	**98.60**	96.71	96.79	99.30
Our model	**97.87**	**98.39**	97.46	97.32	**97.92**	**97.85**	**99.73**

Note: performance values of other methods come from Sharma. The best performance on a metric is marked in bold.

## 4 Discussion

In this paper, we combine CKSAAP, PWAA, N-gram, and raw sequence encoding and apply a deep learning approach to predict AMPs. First, we analyzed the benchmark dataset and compared the differences. Then, we separately evaluated and analyzed the prediction effects of CKSAAP, PWAA, N-gram encoding, and raw sequence encoding combination. Finally, we compare state-of-the-art methods, and the results show that this method has the best performance. We combined CKSAAP, PWAA, N-gram encoding, and original sequence encoding, which not only considered the interaction between amino acids commonly used by other methods, but also considered the upstream and downstream information ignored by other methods, and enhanced the AMPs sequence. Therefore, this method has better performance.

Our method achieves high-precision classification of AMPs based on protein sequence information and yields good performance. But AMPs may have undesirable properties as a drug, including instability and toxicity. In studies of synthesizing and modifying AMPs, even small changes can alter the function of AMPs. This method can only identify AMPs and does not consider the functional characteristics of AMPs. Further research can be carried out according to the functions of AMPs, which will help to better understand the mode of action of AMPs and predict their activities.

## Data Availability

The original contributions presented in the study are included in the article/Supplementary Material, further inquiries can be directed to the corresponding authors.
